# Osteopontin Is a Blood Biomarker for Microglial Activation and Brain Injury in Experimental Hypoxic-Ischemic Encephalopathy

**DOI:** 10.1523/ENEURO.0253-16.2016

**Published:** 2017-01-10

**Authors:** Yikun Li, Eric B. Dammer, Xiaohui Zhang-Brotzge, Scott Chen, Duc M. Duong, Nicholas T. Seyfried, Chia-Yi Kuan, Yu-Yo Sun

**Affiliations:** 1 Department of Pediatrics, Emory University School of Medicine, and Children’s Healthcare of Atlanta, Atlanta, GA 30322; 2 Department of Biochemistry, Emory University School of Medicine, Atlanta, GA 30322

**Keywords:** HIE, hypoxia-ischemia, LC-MS/MS

## Abstract

Clinical management of neonatal hypoxic-ischemic encephalopathy (HIE) suffers from the lack of reliable surrogate marker tests. Proteomic analysis may identify such biomarkers in blood, but there has been no proof-of-principle evidence to support this approach. Here we performed in-gel trypsin digestion of plasma proteins from four groups of 10-d-old mice [untouched and 24 h after low-dose lipopolysaccharide (LPS) exposure, hypoxia-ischemia (HI), or LPS/HI injury; *n* = 3 in each group) followed by liquid chromatography-tandem mass spectrometry and bioinformatics analysis to search for HI- and LPS/HI-associated brain injury biomarkers. This analysis suggested the induction of plasma osteopontin (OPN) by HI and LPS/HI, but not by sham and injury-free LPS exposure. Immunoblot confirmed post-HI induction of OPN protein in brain and blood, whereas *Opn* mRNA was induced in brain but not in blood. This disparity suggests brain-derived plasma OPN after HI injury. Similarly, immunostaining showed the expression of OPN by Iba1^+^ microglia/macrophages in HI-injured brains. Further, intracerebroventricular injection of LPS activated microglia and up-regulated plasma OPN protein. Importantly, the induction of plasma OPN after HI was greater than that of matrix metalloproteinase 9 or glial fibrillary acid protein. Plasma OPN levels at 48 h post-HI also parallel the severity of brain damage at 7-d recovery. Together, these results suggest that OPN may be a prognostic blood biomarker in HIE through monitoring brain microglial activation.

## Significance Statement

There are no reliable blood biomarkers for neonatal brain injury in hypoxic-ischemic encephalopathy (HIE) to date, and the majority of previous studies tested candidate biomarkers individually. Here we report, for the first time, proof-of-principle evidence that mass spectrometry–based quantitative proteomic methods may efficiently identify blood biomarkers in HIE. Using this method, we show that osteopontin (OPN), an integrin-binding protein secreted by activated microglia, rises in blood and correlates with severity of brain damage in experimental HIE. These data suggest that OPN may be a blood biomarker in HIE.

## Introduction

Hypoxic-ischemic encephalopathy (HIE) as a result of perinatal asphyxia is a major cause of neonatal mortality and neurological disabilities, with an incidence of 1–2 per 1000 live births in the Western world and higher incidence in developing countries. Hypothermia is the current best therapy for HIE, but there are no simple laboratory tests to acutely monitor brain damage, which has hindered the development of better treatments (Bennet et al., 2010). Blood biomarkers are an attractive idea to overcome this obstacle, and previous studies using a candidate approach have implicated metalloproteinase 9 (MMP-9) and glial fibrillary acid protein (GFAP) as potential blood biomarkers in HI brain injury (Bembea et al., 2011; Bednarek et al., 2012). However, quantitative proteomics has not been used to identify plasma biomarkers in HIE, despite its success in neurodegenerative disease research (Donovan et al., 2013).

To test the applicability of quantitative proteomics for the discovery of HIE biomarkers, we used in-gel trypsin digestion and liquid chromatography-tandem mass spectrometry (LC-MS/MS) to compare plasma proteins isolated at 24-h recovery from four groups of mouse neonates: unchallenged, treated with low-dose lipopolysaccharide (LPS), Vannucci hypoxia-ischemia (HI), or LPS/HI-injured. The comparison with low-dose LPS-treated animals (which do not exhibit brain injury) is to minimize LPS-induced nonspecific immune alterations in brain damage–related biomarkers in LPS/HI injury. Here we report the proteomics-based discovery and validation of OPN as a plasma biomarker for HIE.

OPN, also called secreted phosphoprotein 1 (SPP1), is a small integrin-binding ligand N-linked glycoprotein (SIBLING) that mediates cell–cell and cell–matrix interactions (Bellahcène et al., 2008). The basal OPN level in adult brains is low, but ischemia induces microglia/macrophages to express OPN in the peri-infarct area (Ellison et al., 1998; Gliem et al., 2015). Previous studies have shown OPN induction in rodent brains after neonatal HI, but whether there is increase of plasma OPN protein has not been evaluated (Chen et al., 2011; van Velthoven et al., 2011). Earlier studies suggested protective functions of OPN in neonatal HI, but later studies failed to confirm this finding (Albertsson et al., 2014; Bonestroo et al., 2015). Although the role of OPN in HI-injured brains remains uncertain, our results implicate plasma OPN as a prognostic biomarker for microglia activation and brain damage in HIE.

## Materials and Methods

### Animal surgery

The Vannucci model of neonatal HI with and without LPS preexposure (0.3 mg**/**kg, IP, 4 h pre-HI), and intracerebroventricular (ICV) injection of LPS (1 μg) was performed as described (Yang et al., 2009, 2014). Briefly, 10-d-old C57BL/6 mice of both sexes were subjected to ligation of the right common carotid artery and, 1 h later, exposure to 10% O_2_ for 40 min in glass chambers submerged in a 37°C water bath. These procedures were approved by the Institutional Animal Care and Use Committee and conformed to the NIH Guide for Care and Use of Laboratory Animals.

### Plasma preparation and proteomic analysis

Blood, collected from rodent neonates into Eppendorf tubes containing EDTA, was centrifuged at 1000 × *g* for 15 min. Supernatants were resolved by SDS-PAGE for proteomic analysis. Five gel slices for each sample were excised based on molecular weight and individually digested with trypsin (12.5 ng/µL), and the resulting peptides were analyzed independently by reverse-phase liquid chromatography coupled with tandem mass spectrometry on an Orbitrap-XL mass spectrometer (Thermo Fisher Scientific) essentially as previously described (Dammer et al., 2013, 2015). MS/MS spectra were searched against a concatenated target-decoy mouse NCBI reference database using the SEQUEST Sorcerer algorithm (version 4.3.0, SAGE-N). Peptides were classified by charge state and filtered by mass accuracy (±10 ppm), and then dynamically by increasing cross-correlation (XCorr) and delta correlation (ΔCn) values to reduce protein false discovery rate to <1%. Peptide-specific ion current intensities were extracted and compared using in-house software (Herskowitz et al., 2011; Donovan et al., 2013). Accurate peptide mass and retention time were used to derive signal intensity for every peptide across LC-MS runs for each sample. Only high-confidence differential proteins [log_2_(ratio) ≥ ±0.89; signal-to-noise ratio > 5 compared with controls] with ≥2 MS/MS-identified peptide counts were tabulated.

### Multiplex assays using Luminex

The Luminex bead-based multiplex ELISA was designed following the manufacturer’s instructions (R&D Systems). Reactions were run on a Bio-Plex Multiplex System, and data were analyzed using Bio-Plex Manager Software.

### Quantitative RT-PCR

RNA was isolated using the TRIzol RNA isolation kit (Invitrogen) from rat and mouse peripheral blood mononuclear cells, microglia SM826 cells, and neonatal brain tissues. Reverse transcription was performed following the manufacturer’s instructions (Applied Biosystems). Quantitative real-time PCR was performed using a CFX 96 system (Bio-Rad) and detected by SYBR Green master mix (Bio-Rad) as previously described (Yang et al., 2014). The following *Opn* primers were used for PCR reaction: rat, GCTCTCAAGGTCATCCCAGTTG; TGTTTCCACGCTTGGTTCATC; mouse, AGCCACAAGTTTCACAGCCACAAGG; CTGAGAAATGAGCAGTTAGTATTCCTGC.

### Antibodies

The following antibodies were used for immunoblotting or immunofluorescence labeling: anti-OPN (R&D Systems, AF808), anti–cystatin C (R&D Systems, AF1238), anti-GAPDH (EMD Millipore, MAB374), anti–β-actin (Sigma-Aldrich, A5441), anti-MMP9 (Sigma-Aldrich, AV33090), anti-GFAP (EMD Millipore, AB5804), anti-Iba1 (Wako, 019-1974), anti-transferrin (Abcam, ab9538), anti-NG2 (EMD Millipore, AB5320), and anti-NeuN (EMD Millipore, MAB377). Secondary antibodies for immunocytochemistry were conjugated to Alexa Fluor 488 and 594 (Invitrogen).

### Statistical analyses

All values are expressed as mean ± SD. Significance of correlations was determined by Pearson’s correlation coefficient (GraphPad Prism). *p* < 0.05 was considered significant. Reporting of significantly changed proteins identified by label-free quantitative proteomics relied on *p* < 0.01 (confidence interval 99%), and the empirical false-positive rate was determined as previously reported (Donovan et al., 2013; Dammer et al., 2015), in a null experimental comparison of controls, to be in the range of 10%–15%.

## Results

### HI and LPS/HI insults, but not systemic LPS exposure, elevate plasma OPN levels

**Figure 1.**
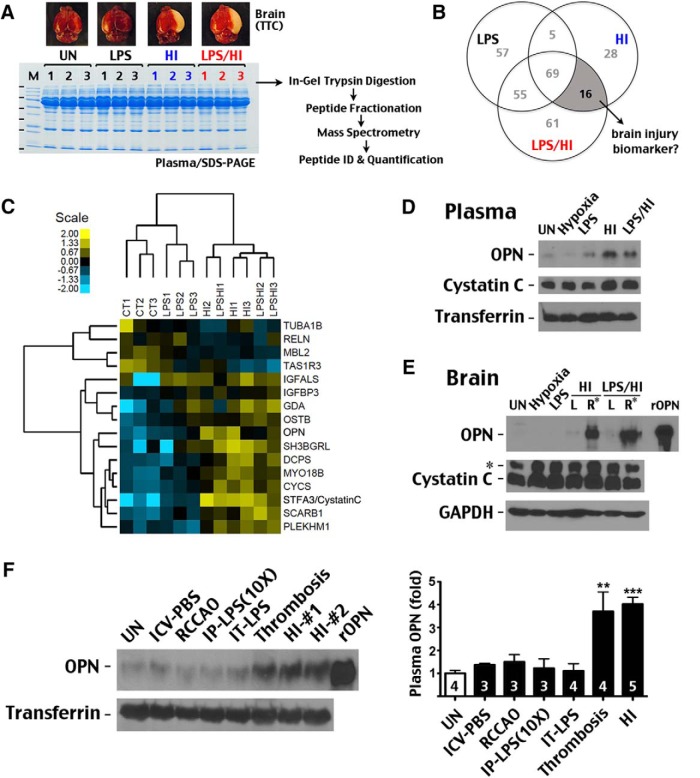
Proteomic analysis of plasma biomarkers for murine neonatal HI brain injury. ***A***, Plasma proteins collected from unchallenged (UN), low-dose LPS (0.3 mg/kg, IP)-exposed, and HI- or LPS/HI-injured P11 mice were separated by SDS-PAGE and analyzed as depicted (*n* = 3 for each). Also shown are representative tetrazolium chloride–stained brains in each group, showing cerebral infarction (pale color) in only HI- or LPS/HI-injured animals. ***B***, Venn diagram showing the number of unique proteins in the depicted conditions compared with the unchallenged animals. ***C***, Heatmap and hierarchical clustering of the 12 examined animals based on the signal intensity of HI- and LPS/HI-associated proteins. Shown is the log_2_ scale of fold change. ***D***, ***E***, Immunoblot showing specific OPN induction in plasma and the ipsilateral hemisphere (right, R^*^) of mice after HI or LPS/HI insult. In contrast, although cystatin C showed increased plasma levels after HI and LPS/HI injury, it was not elevated in the brain after these injuries. *, Nonspecific anti–cystatin C band in the brain. rOPN, recombinant mouse OPN used as positive control. ***F***, Immunoblotting showing that cerebral stroke (thrombosis) also induced high levels of plasma OPN, whereas ICV injection of phosphate saline (ICV-PBS), unilateral carotid artery ligation (RCCAO), i.p. injection of 3 mg/kg LPS (IP-LPS[10×]), and intratracheal application of 0/3 mg/kg LPS (IT-LPS) lacked this effect. The numbers of mouse pups examined in each condition are indicated. **, *p* < 0.01; ***, *p* < 0.001 by *t*-test compared with unchallenged animals.

Plasma proteins of four groups of postnatal day 11 (P11) C57BL/6 mice of both sexes, unchallenged or 24 h after LPS exposure (0.3 mg/kg, i.p.), pure HI, or LPS/HI insult (*n* = 3 in each group), were resolved by SDS-PAGE followed by in-gel trypsin digestion and LC-MS/MS analysis (Fig. 1*A*). Tetrazolium chloride staining confirmed cerebral infarct after HI and LPS/HI injury (pale color in Fig. 1*A*), but not after low-dose LPS exposure. Label-free quantification was performed based on ion current measurements of identified peptides. By using fold change [log_2_(ratio) ≥ ±0.89] and peptide spectral count (≥2) cutoffs, we identified 16 proteins that were uniquely associated with HI and LPS/HI insults, but not with LPS exposure, compared with the unchallenged plasma samples (Fig. 1*B*, *C*). Among the 16 candidate biomarkers, OPN increased 3.2-fold after HI and 2.5-fold after LPS/HI injury (Table 1). Cystatin C/stefin-3 (STFA3), which increased 6.2-fold after HI and 3.2-fold after LPS/HI, is another intriguing candidate, since it was suggested to be a biomarker for renal injury in neonatal asphyxia (Allegaert et al., 2015). Thus, we attempted to validate these two potential blood biomarkers; the prognostic value of the other candidates can be investigated in future studies.

**Table 1.** Plasma proteins that are uniquely associated with HI and LPS/HI brain injury at 24-h recovery

Symbol	Description (all in *Mus musculus*)	Peptide feature counts	CT/CT log2(ratio)	CT/CT SD, *n* = 3	LPS/CT log2(ratio)	LPS/CT SD, *n* = 9	HI/CT log2(ratio)	HI/CT SD, *n* = 9	LPS/HI/CT log2(ratio)	LPS/HI/CT SD, *n* = 9
TUBA1B	Tubulin alpha-1B chain	58	–0.6	0.33	–0.78	0.5	–0.94	0.47	–1.11	0.41
RELN	Reelin precursor	18	0.01	0.22	–0.57	0.35	–0.96	0.27	–1.05	0.14
MBL2	Mannos-binding protein C precursor	86	0.23	0.28	–0.51	0.43	–1.17	0.3	–1.55	0.3
TAS1R3	Taste receptor type 1 member 3 precursor	5	–0.35	0.17	–0.77	0.43	–1.22	0.53	–1.38	0.56
IGFALS	Insulin-like growth factor-binding protein complex acid labile subunit precursor	171	–0.11	0.26	–0.56	0.18	–1.78	0.34	–2.06	0.31
IGFBP3	Insulin-like growth factor-binding protein 3 precursor	27	–0.19	0.16	0.13	0.16	–0.92	0.25	–1.28	0.35
GDA	Guanine deaminase	15	0.25	0.36	0.62	0.34	1.03	0.45	1.05	0.49
OSTB	Organic solute transporter subunit beta	8	0.42	0.14	0.79	0.29	0.9	0.31	1.05	0.33
OPN	Osteopontin	11	0.12	0.4	0.69	0.28	1.68	0.6	1.31	0.43
STFA3	Stefin-3 (cystatin C)	14	–0.48	0.99	0.62	0.68	2.63	0.71	1.67	0.6
MYO18B	Myosin XVIIIb	2	–0.25	0.15	0.8	0.25	1.51	0.31	1.3	0.2
SH3BGRL	SH3 domain-binding glutamic acid-rich like protein	3	0.33	1.21	–0.09	1.98	1.86	0.84	1.7	0.76
DCPS	m7GpppX diphosphatase	12	0.2	0.35	0.34	0.45	1.55	0.61	1.47	0.32
PLEKHM1	Pleckstrin homology domain-containing family M member 1	2	–0.05	0.49	–0.12	0.45	0.92	0.42	1.06	0.44
CYCS	Cytochrome c, somatic	50	–0.35	0.14	0.64	0.21	1.59	0.23	1.44	0.28
SCARB1	Scavenger receptor class B member 1 isoform 1	2	–0.25	0.1	0.71	0.25	1.3	0.4	1.75	0.46

Plasma proteins associated with HI and LPS/HI brain injury at 24-h recovery in P10 mice. Proteomics detection and statistical analysis were performed as described in the text. Sixteen high-confidence proteins changing in this category with ≥2 MS/MS-identified peptide feature counts used for quantitation across the 12 samples are reported.

To confirm the proteomic findings, we used immunoblotting to examine OPN and cystatin C in blood and brain 24 h after hypoxia, LPS exposure, HI, or LPS/HI insult (Fig. 1*D*, *E*). This analysis validated an increase of both OPN and cystatin C in the blood after HI- and LPS/HI injury, but we chose to further characterize OPN because the basal level of plasma OPN is very low, making its induction more unequivocal. Moreover, HI and LPS/HI induce robust OPN expression in the ipsilateral cortex (right; R* in Fig. 1*E*), raising the possibility that plasma OPN may derive from the HI-injured brain.

To test its specificity as a biomarker, we compared the amount of plasma OPN 24 h after ICV injection of phosphate saline, ligation of the right common carotid artery, i.p. injection of high-dose (3 mg/kg) LPS, intratrachea injection of 0.3 mg/kg LPS, cortical photothrombosis, and HI injury in P10 mice (Fig. 1*F*; numbers of mice used for each condition are indicated). The analysis showed that only thrombosis and HI caused significant induction of plasma OPN (**p < 0.01; ***p < 0.001 compared with basal level in unchallenged mice). These data suggest that plasma OPN may monitor a common pathological process following cerebral ischemia and HI, such as microglia activation, but is not a sensitive marker for systemic immune activation.

### HI-induced plasma OPN is derived from brain microglia/macrophages

**Figure 2.**
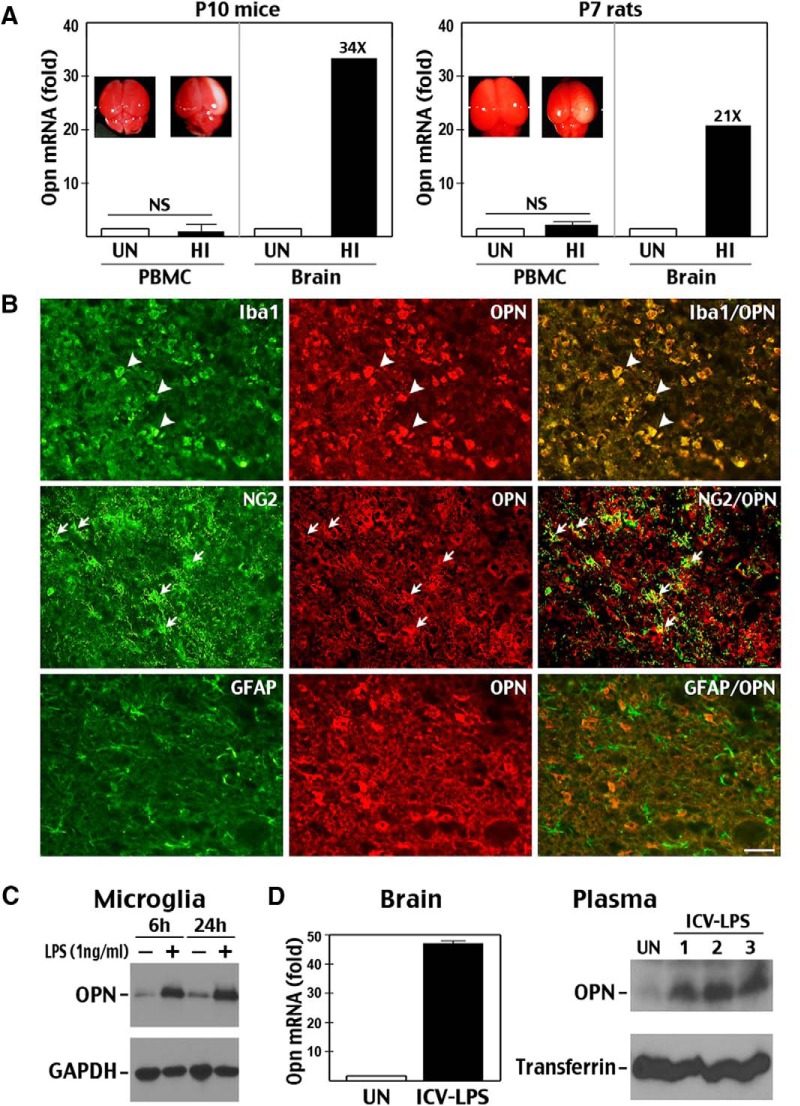
Source and specificity of plasma OPN as a biomarker. ***A***, RT-PCR showed that HI triggered a 20- to 30-fold increase of *Opn* mRNA in the brain of P10 mice and P7 rats at 24-h recovery, but only insignificant changes in PBMCs (*n* = 4–5 for each group). ***B***, Immunolabeling showed widespread OPN expression by Iba1^+^ microglia and occasional expression in NG2^+^ glial progenitors, but rare expression in GFAP^+^ astrocytes after HI injury (*n* > 4). Scale bar: 40 μm. ***C***, Application of LPS (1 ng/ml) to microglial SM826 cells in vitro led to induction of OPN protein. ***D***, Similarly, ICV injection of LPS (1 μg) induced *Opn* mRNA in brain as well as plasma OPN protein at 24-h recovery.

To test the source of plasma OPN, we compared *Opn* mRNA levels in brain and peripheral blood mononuclear cells (PBMCs) after HI insult in mouse and rat neonates. The analysis showed 20- to 35-fold increases of *Opn* mRNA in brain, but minimal changes in PBMCs in animals that exhibited cerebral infarction 24 h post-HI (Fig. 2*A* and insets). Our data are consistent with past studies showing strong post-stroke induction of *Opn* mRNA in rodent brain, but not in blood (Tang et al., 2002, 2003).

To determine OPN-expressing cells, we used immunostaining to examine HI-injured brains and found strong OPN expression in Iba1^+^ microglia/macrophages and occasional OPN expression in NG2^+^ glial progenitors, but not in GFAP^+^ astrocytes (Fig. 2*B*), similar to previous reports (Ellison et al., 1998; Gliem et al., 2015). Accordingly, *in vitro* LPS stimulation triggered the expression of OPN by microglial cells (Fig. 2*C*). Moreover, ICV injection of LPS, a model of selective microglia activation, induced brain *Opn* mRNA and plasma OPN protein 24 h later (Fig. 2*D*). These results suggest that OPN is mainly produced by activated microglia and macrophages in HI-injured brain and is subsequently transported to blood as a secreted phosphoprotein.

### Plasma OPN is a sensitive and predictive biomarker for brain injury in experimental HIE

**Figure 3.**
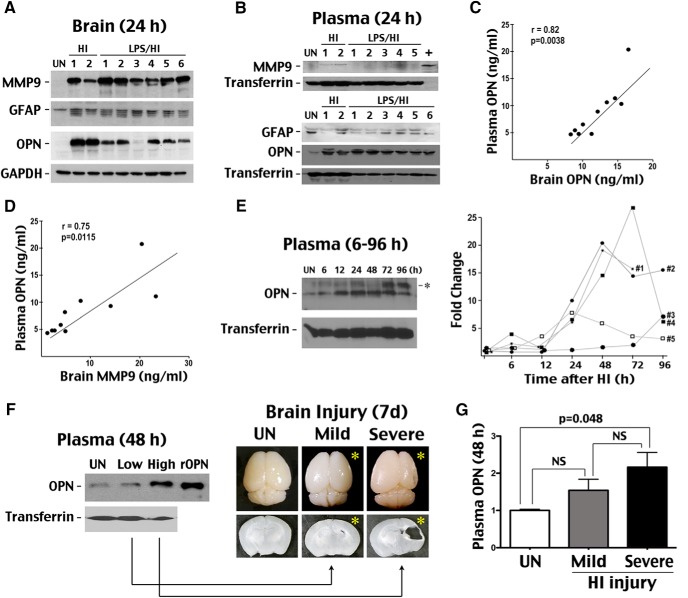
Sensitivity and prognostic value of plasma OPN as a biomarker for HI brain injury. ***A***, ***B***, Comparison of OPN and two other candidate biomarkers (MMP-9 and GFAP) in brain and blood after HI and LPS/HI injury. In brain extracts, elevation of MMP-9 or OPN expression and multiple GFAP bands were all observed after injury, but in plasma, only OPN induction was detectable at 24 h post-insult. ***C***, ***D***, Multiplex ELISA was used to compare brain and plasma OPN or MMP-9 levels in P10 mice after HI. Plasma OPN levels showed strong correlation with brain OPN (*r* = 0.82) and brain MMP-9 levels (*r* = 0.75, *n* = 10) 24 h after HI injury. ***E***, HI-injured P7 rats were used to assess the time course of plasma OPN induction after HI injury (*n* = 5). Strong (>5-fold) induction of plasma OPN occurred only 24 h post-HI. *, Nonspecific signal or hyperphosphorylated OPN. ***F***, Comparison of plasma OPN levels at 48 h post-HI to severity of brain injury at 7 d. Shown are representative subjects for the low and high plasma OPN groups. *, HI-injured hemisphere. ***G***, Quantification showed significant elevation of plasma OPN levels in the severe damage group compared with unchallenged mice (*n* = 5 and 6 per group for mild and severe brain injury, respectively; *p* = 0.048 by *t*-test).

We also compared protein levels of OPN, MMP-9, and GFAP in brain versus blood after HI in murine neonates. Immunoblotting suggested that the presence of multiple GFAP bands and the increase of MMP-9 or OPN are all sensitive markers of HI injury in the brain (Fig. 3*A*). In contrast, only induction of OPN, but not MMP-9 or GFAP, was clearly detectable in the blood of these HI-injured animals at 24 h recovery (Fig. 3*B*). Moreover, multiplex ELISA (Luminex) showed strong correlation between plasma OPN levels and OPN (Fig. 3*C*; *p* = 0.0038) and MMP-9 (Fig. 3*D*; *p* = 0.0115; *n* = 10) levels in the brain at 24-h recovery. These results suggest that plasma OPN is a monitoring biomarker for brain damage in experimental HI.

To examine the timing of plasma OPN induction after HI, we collected serial blood samples at 6, 12, 24, 48, 72, and 96 h postinjury from a cohort of postnatal day 7 (P7) rats for immunoblotting. This analysis showed variable dynamics of OPN alterations in HI-injured animals, but strong induction (>5-fold) occurred only 24 h post-HI (Fig. 3*E*; *n* = 5). These data suggest that the increase of plasma OPN may indicate the minimal onset time of perinatal asphyxia.

Finally, to test the prognostic value of plasma OPN for HI brain damage, we shortened the duration of hypoxia (to 35 min) to produce more variable brain damage in the same cohort of mouse pups (*n* = 11) and examined the relationship between the severity of brain injury at 7 d post-HI and the corresponding plasma OPN levels at 48 h (Fig. 3*F*). We found that mice with mild brain injury (*n* = 5) exhibited low plasma OPN levels that were indistinguishable from baseline at 48 h post-HI, whereas animals with severe brain damage (*n* = 6) showed higher plasma OPN levels than baseline at 48 h post-HI (Fig. 3*G*). The 48 h plasma OPN levels between mice that would later develop mild- or severe- brain damage were not significantly different, however, owing to outliers in each group. These results suggest that plasma OPN levels have predictive value for the severity of HIE brain injury, but multiple time-point monitoring or the use of additional biomarkers is needed to increase prognostic accuracy.

## Discussion

The objective of this study is to test the applicability of quantitative proteomic methods toward the discovery of blood biomarkers for HI brain injury. Although therapeutic hypothermia (to 32–34°C) starting within 6 h after HI insult and continuing for 12–72 h improves outcomes, 40%–50% of infants treated with hypothermia still die or develop significant neurological disabilities. Thus, there is a need for better HIE therapies and reliable biomarkers to monitor response to treatment (Bennet et al., 2010). A recent study indicated that magnetic resonance imaging performed at 44 weeks postmenstrual age or when clinically feasible is a reliable indicator of neurodevelopmental outcomes at 6–7 y of age (Shankaran et al., 2015). Yet simple laboratory assays using blood or urine that can be performed more widely and acutely remain a highly useful tool in clinical care of HIE. To this end, past research using a candidate approach has suggested several blood biomarkers in HIE, such as GFAP and MMP-9 (Bembea et al., 2011; Bednarek et al., 2012). In contrast, an open-ended proteomic strategy has not been used, despite its application in identifying blood biomarkers in obstetrical conditions (Klein et al., 2014). In preclinical study, only 2D differential gel electrophoresis has been used to compare brain proteins after HI (Rosenkranz et al., 2012). To our knowledge, the present study is the first report using quantitative proteomics to identify blood biomarkers in experimental HI. Our results support the use of quantitative proteomics for biomarker discovery and suggest osteopontin as a prognostic blood biomarker in HI brain injury.

In our experimental design, the control group of low-dose i.p. LPS exposure (which does not produce obvious brain damage) is designed to avoid non–lesion-associated immune alterations in LPS/HI injury. Using MS and statistical analysis of the plasma proteins in four experimental groups, we revealed 16 proteins that are associated with both HI and LPS/HI brain injury. Among them, we selected OPN and cystatin C for validation, given their relevance to neonatal asphyxia in the literature, and saved the other candidates for future study. Specifically, the plasma cystatin C level has been suggested to be a biomarker of renal injury in neonatal asphyxia (Allegaert et al., 2015). Induction of protein and mRNA of OPN was also reported in HI-injured rodent neonatal brains (Chen et al., 2011; van Velthoven et al., 2011).

Our immunoblot results validated the increase of both OPN and Cystatin C in blood after HI and LPS/HI injury, but we decided to further characterize OPN given its low basal level (which assists the detection of postinjury induction) and lesion-side specific induction in HI- and LPS/HI-injured brains. These characteristics raise the possibility that post-HI plasma OPN may derive from the injured brain, a scenario that is further supported by two observations. First, we showed that HI induces *Opn* mRNA in brain, but not in blood, consistent with a previous finding of brain-specific induction of *Opn* mRNAs in cerebral ischemia models (Tang et al., 2002, 2003). Second, cerebral ventricular injection of LPS, a model of selective microglia activation, was able to elevate plasma OPN levels, whereas i.p. injection of 10-fold, 3-mg/kg LPS, a model to induce systemic immune responses, lacked this effect.

In addition, our results suggest that plasma OPN is more sensitive than MMP-9 and GFAP as a biomarker of experimental HI brain injury. The higher sensitivity of OPN after HI injury may relate to its intrinsic property as a secreted protein or its greater stability in blood (Lanteri et al., 2012). We also showed that plasma OPN induction typically occurs after 24 h of HI onset and closely correlates with brain MMP-9 levels, and that high levels of plasma OPN at 48 h post-HI correlate with severe brain injury at 7 d. Whether human HIE neonates show similar induction of plasma OPN warrants further investigation. If a similar pattern is observed, monitoring plasma OPN levels in neonates may serve two purposes. First, an increase of plasma OPN may indicate the minimal onset time of perinatal HI (∼24 h), which would suggest intrauterine HI stress and poorer response to hypothermia treatment. Second, the trajectory of plasma OPN alterations may indicate the progression or recovery of brain injury in postasphyxia neonates. Importantly, our results do not exclude the possibility that plasma OPN may also derive from activated macrophages outside the brain. Hence, differential diagnosis and multiple-tissue monitoring remain essential for the interpretation of plasma OPN induction in neonatal care.

MS-based proteomics is a powerful strategy to determine disease-associated biomarkers in biofluids, including blood, urine, and cerebrospinal fluid. With this methodology, no additional *a priori* hypothesis of the biomarker is needed, and once a protein biomarker is found, simple and relatively cheap laboratory tests can be developed for multiple time-point monitoring of disease progression, even in less-developed areas of the world. Moreover, when applied to well-controlled samples, quantitative proteomics is a very efficient method to uncover differential biomarkers. For example, the present study used only four experimental groups and three mice per group, but we were able to uncover OPN as a blood biomarker of HI brain injury by statistical analysis. By adding comparison groups and enlarging the sample size, this quantitative proteomic strategy may reveal additional blood biomarkers in HIE.

In summary, our findings support the application of proteomic analysis to identify biomarkers in HIE. Moreover, the prognostic value of plasma OPN in HI brain injury warrants investigation in human infants.

## Synthesis

Reviewing Editor: Jun Chen, University of Pittsburgh Decisions are customarily a result of the Reviewing Editor and the peer reviewers coming together and discussing their recommendations until a consensus is reached. When revisions are invited, a fact-based synthesis statement explaining their decision and outlining what is needed to prepare a revision will be listed below. The following reviewer(s) agreed to reveal their identity: Hitoshi Komuro.

The current submission explores the use of quantitative proteomics to explore plasma biomarkers associated with neonatal hypoxic-ischemic encephalopathy. The need for biomarkers validates the intent of the study, and would be highly significant in clinical settings. The authors identify osteopontin as a potential candidate. The increase in OPN protein in both brain and blood after HI and LPS/HI was confirmed by immunoblotting. Notably, HI treatment increased the Opn mRNAs in the brain, but not in blood, suggesting a brain origin of plasma OPN after HI. The plasma OPN levels were highly correlated with the brain OPN at 24 h after HI, and the elevations of plasma OPN were more sensitive than glial fibrillary acid protein (GFAP) and matrix metalloproteinase after HI injury. Furthermore, the use of immunostaining showed that OPN-expressing cells in HI-injured brains are Iba1-positive microglia/macrophages, but not GFAP-positive astrocytes, indicating that upon HI brain injury, microglia/macrophages produce OPN, which is in turn released to the blood. In conclusion, these results suggest that plasma OPN may be a sensitive biomarker of HIE brain injury.

Both reviewers acknowledged that the results of the current study are very interesting, that the study was well planned and organized, and that the observations reported in this manuscript should have potential clinical implications. However, both reviewers indicated that some surgical controls were missing, which should be included. Below lists the specific comments from the reviewers:

1. (Major) Surgical controls, including sham operations for the ischemic induction as well as sham icv injection, are needed, particularly when using the Vannucci model, in part due to the presence of anesthesia. Inclusion of sham surgical control groups with and without hypoxia would also address whether OPN is sensitive to hypoxia exposure (a small piece of data regarding this is included in the hemispheric immunoblotting in Fig. 1E, but would be useful to know if this sort of "control" hemisphere was used elsewhere in the study). Inclusion of details on the HIE procedure would be helpful (e.g., temperature, the reduction of HI duration (Fig. 3G)), rather than just a citation.

2. (Major) The following issues should be carefully considered and discussed or clarified:

(1) One issue lies in the use multiple LPS paradigms used. Administration routes vary between between figure subsets (systemic vs local, and intraparitoneal vs. intracerebroventricular vs intratracheal). The functional nuances on gene expression between systemic LPS delivery and local LPS delivery should be carefully considered and well expressed within the text regarding the rational for these delivery modes and the changes in doses. How are they comparable?

(2) Similarly, the authors state that the "low dose LPS-treatment group is used to exclude non-specific immune alterations in LPS/HI injury." However, low dose LPS alone may act biologically different than low dose LPS with HI, in that low dose LPS may induce a preconditioned state that the combination of LPS and HI may not permit. Preconditioning may elicit differential patterns of gene expression that cannot really be then used as an appropriate control for exclusion of "non-specific" genes.

(3) The authors state in Fig. 1B that OPN is not in the shaded region - a region that specifically excludes LPS-only gene induction. Additionally, the single immunoblot show for Fig. 2E suggests that high dose LPS cannot robustly induce LPS. However, in Fig. 2D, the authors show that local (icv) injection of LPS effectively increased OPN gene expression. Although the delivery routes and dosages are different, OPN then may not be exclusive for HI conditions. This discrepancy needs to be addressed.

(4) The labeling of Fig. 4G as "mild" vs "severe" HI injury is a little misleading, as it implies that post hoc histological assessments were used to define the injury. In fact, the model itself was altered to have reduced duration. Instead of labeling "mild" vs. "severe", these bars should be labeled with the time duration of HI. A post hoc histological assessment correlated with plasma OPN values would be a better argument for the predictive capacity of OPN as a biomarker.

3. (Minor) There are a number of minor issues that need to be addressed:

(1) There is no indication on the number of animals used for Fig. 3G.

(2) If Fig. 2E using >10 samples shown, semi-quantification of the immunoblots would be useful to illustrate the reported results in terms of consistency of OPN induction. Semi-quantification of other blots in Fig. 3 would also be useful.

(3) Table1 shows that SH3BGRL and DCPS are also potential candidates for sensitive biomarkers of HIE brain injury. The authors may add some comments about SH3BGRL and DCPS in the discussion section.

(4) In some occasions, the increases of plasma OPN may be induced by injury or damage of tissues other than brain. The authors may explain such cases in the discussion section.

(5) HI may cause the activation of NG2-positive cells in brains. My question is whether the activated NG2-positive cells express OPN. The authors may explain this possibility in the discussion section.
